# Stricter Teacher, More Motivated Students? Comparing the Associations Between Teacher Behaviors and Motivational Beliefs of Western and East Asian Learners

**DOI:** 10.3389/fpsyg.2020.564327

**Published:** 2021-01-15

**Authors:** Yushan Jiang, Chi-Kin John Lee, Zhi Hong Wan, Junjun Chen

**Affiliations:** ^1^Department of Curriculum and Instruction, The Education University of Hong Kong, Tai Po, Hong Kong; ^2^Department of Education Policy and Leadership, The Education University of Hong Kong, Tai Po, Hong Kong

**Keywords:** teacher feedback, teacher strictness, motivational beliefs, Western and East Asian learners, interpersonal behavior and communication, teacher behavior and classroom practice

## Abstract

Teacher behaviors are one of the most significant factors influencing student learning. Students from different cultures may have different interpretations of their teachers’ behaviors. This study compared the associations between teacher strictness, teacher feedback, and students’ motivational beliefs using data from six Western countries (the United States, the United Kingdom, Finland, Norway, Australia, and New Zealand) and six East Asian regions (Japan, Korea, mainland China, Hong Kong, Macau, and Taiwan) in the Program for International Student Assessment (PISA) 2015. A total of 89,869 15-year-old students were included in data analysis. The findings indicate that (i) teacher strictness was negatively associated with Western students’ motivation, but positively related to that of East Asian students; (ii) teacher feedback had significant positive associations with the motivational beliefs of both Western and East Asian students; and (iii) there was a positive relationship between teacher strictness and teacher feedback in East Asian context. These results highlight the need to consider cultural factors when interpreting students’ reactions to teacher behaviors.

## Introduction

In the past few decades, teacher behaviors have attracted considerable attention in the fields of learning environment and educational effectiveness (e.g., den Brok et al., 2004; Kyriakides et al., 2020). A growing body of studies have revealed the significant influence of teacher behaviors on students’ engagement, motivation, and achievement (Brekelmans et al., 2000, 2002; Roorda et al., 2011; Wubbels et al., 2016).

Teacher behaviors can be broadly classified as interpersonal and teaching behaviors. Interpersonal behaviors are usually conceptualized and investigated using the Model of Interpersonal Teacher Behavior (MITB; Wubbels and Brekelmans, 2005), which encompasses eight sectors of behaviors, namely, leadership, helpful/friendly, understanding, student freedom, uncertainty, dissatisfaction, and strictness. Previous empirical studies showed that teachers’ favorable interpersonal behaviors are strongly correlated with student motivation (e.g., den Brok et al., 2004; Lapointe et al., 2005). Maulana et al. (2011) found high student motivation was moderately related to teachers’ proximity and influence behaviors with Indonesian samples. Likewise, van Uden et al. (2014) reported positive correlations between student engagement and teachers’ influence and proximity in the Dutch context. In addition, teacher’s interpersonal behaviors have been identified to influence students’ learning attitudes (Quek et al., 2007).

As for teaching behaviors, previous studies have investigated the impacts of feedback, clarity, modeling, questioning, reinforcement, and communication of teacher expectations on student learning (e.g., Creemers, 1994; Soh, 2017; Gentrup et al., 2020; Kyriakides et al., 2020). Among these constructs, teacher feedback has been considered as one of the most important practices for improving student learning (Gentrup et al., 2020). Previous research has revealed a direct positive impact of teacher feedback on students’ self-efficacy (Rakoczy et al., 2019) and motivation (e.g., Hamidun et al., 2012; Pat-El et al., 2012). In particular, scaffolding behaviors in the form of giving extra information about how to improve performance on tasks have shown to have a positive influence on student motivation (Dresel and Haugwitz, 2008).

Although previous studies have revealed a close relationship between teacher behaviors and student learning, most of these studies were conducted in the West (Pennings et al., 2014; Pennings and Hollenstein, 2019; Sun et al., 2019). However, as revealed in some comparative studies, teacher behaviors might be interpreted differently by East Asian and Western learners. For example, Chinese students in Lewis et al.’s (2008) study believed their teachers’ disciplinary actions are more justified when compared to Australian students. Zhou et al. (2012) have found that Chinese students perceived less controls when their teachers provide corrective feedbacks, while their American counterparts perceived more controls. Given the differences between East Asian and Western learners in their interpretations of same teacher behaviors, their motivational and behavioral reactions to same teacher behaviors might also differ (Tsai et al., 2016). In other words, there may exist cultural differences in the relationship between teacher behaviors and student learning. To date, there is a scarcity of research to explore such cultural differences.

This study aims to explore the associations between two kinds of teacher behaviors (i.e., teacher strictness and teacher feedback) and four motivational beliefs (i.e., intrinsic motivation, instrumental motivation, achievement motivation, and self-efficacy) of Western and East Asian learners. *Intrinsic motivation* refers to the enjoyment and interests that students may experience from the learning process, *instrumental motivation* is the perceived usefulness of learning in students’ future studies and career, and *achievement motivation* encompasses students’ needs for success and excellence (Cheng and Wan, 2016). In addition, *self-efficacy* refers to students’ beliefs in the extent to which s/he will perform well in a task (Wang et al., 2014). Together, these motivational beliefs are strong predictors of students learning and achievement.

Current study utilized PISA 2015 data to explore the cultural difference between Western and East Asian learners for two reasons: (i) PISA utilizes standardized tools across the Organization for Economic Cooperation and Development (OECD) countries, providing an opportunity to make fair comparisons; and (ii) it adopts a very strict sampling procedure that enables accurate statistical analyses. Therefore, it might be meaningful to explore whether there are significant correlations between teacher strictness and teacher feedback in this study and whether their relationship is also culturally embedded. In sum, the following questions were investigated in this study:

1. How do teacher strictness and teacher feedback affect the motivational beliefs of Western and East Asian learners?
2. How do teacher strictness and teacher feedback correlate in Western and East Asian contexts?

## Methods

### Data

The empirical analysis in this study relies on the PISA 2015 data downloaded in January 2020. Approximately 540,000 15-year-old students from 72 countries and economies were asked to fill out questionnaires and assessments to evaluate their attitudes, motivation, and academic performance (OECD, 2016). The database is publicly available at the OECD website^1^.

To ensure cross-cultural investigation, 12 countries and economies were selected for this study. Six were Western countries (i.e., the United States, the United Kingdom, Finland, Norway, Australia, and New Zealand) and six were East Asian countries or economies (i.e., Japan, Korea, mainland China, Hong Kong, Macau, and Taiwan). The study data came from 89,869 students from the selected countries and regions. Of these students, 44,149 (49.1%) were girls and 45,720 (50.9%) were boys; 50,257 (55.9%) were from the West and 39,612 (44.1%) were from the East Asia.

### Variables

The PISA 2015 included two scales related to teachers’ behavior (teacher feedback and teacher strictness) and four scales related to students’ motivational beliefs (intrinsic motivation, instrumental motivation, achievement motivation, and self-efficacy). The questionnaires can be accessed through the PISA website^2^, and the items of measurements for current study are illustrated in Table A1.

#### Teacher Strictness

This index included four items that capture students’ perceptions of the ways their teachers treat them. Sample items are “Teachers disciplined me more harshly than other students.” and “Teachers graded me harder than they graded other students.” The four-point Likert scale was adopted with 1 indicating “never or almost never,” 2 indicating “a few times a year,” 3 indicating “a few times a month,” and 4 indicating “once a week or more.”

#### Teacher Feedback

This index included five items that capture students’ perception of the frequency of receiving teacher’s formative feedback. Sample items include “The teacher tells me how I am performing in this course.”; “The teacher tells me in which areas I can still improve.” The four-point Likert scale was used with 1 indicating “never or almost never,” 2 indicating “some lessons,” 3 indicating “many lessons,” and 4 indicating “every lesson or almost every lesson.”

#### Motivational Beliefs

As the focus of PISA 2015 was science subjects, the structure and design of the questionnaire was specifically targeted at students’ science learning. Students’ motivational beliefs were measured through: intrinsic motivation (5 items), instrumental motivation (4 items), achievement motivation (5 items), and science self-efficacy (8 items). Measures of intrinsic and instrumental motivation and self-efficacy assessed students’ motivational beliefs within a science learning context, whereas achievement motivation assessed students’ overall motivation. Sample items are “I am interested in learning about science,” “Many things I learn in my science subject(s) will help me to get a job,” and “I want to be the best, whatever I do,” respectively, for intrinsic, instrumental, and achievement motivation. In order to ensure consistency in construct scaling, the responses for instrumental motivation have been reverse coded, so that 1 indicates strongly disagree and 4 indicates strongly agree for all three motivation measurements. For science self-efficacy, the students were asked to rate their confidence in completing particular science-related tasks, such as “Explain why earthquakes occur more frequently in some areas than in others.” Responses were reverse coded on the four-point scale with 1 being “I could not do this,” 2 being “I would struggle to do this on my own,” 3 being “I could do this with a bit of effort,” and 4 being “I could do this easily.”

### Data Analysis

Cronbach’s alpha was calculated for the six scales in the survey as an indicator of their reliability (Table 1). The overall alpha coefficient for teacher strictness was 0.718, and the overall alpha coefficient for teacher feedback was 0.932. For constructs under motivational beliefs, the alpha coefficients ranged from 0.848 to 0.951. As suggested by Fink (2015), the criterion for Cronbach’s alpha coefficient is 0.70, therefore all these scales can be considered to have a good reliability.

**TABLE 1:** Alpha coefficients for the six constructs.

Constructs	Alpha coefficients
Teacher behavior	
Teacher strictness	0.718
Teacher feedback	0.932
Motivational beliefs	
Intrinsic motivation	0.951
Instrumental motivation	0.935
Achievement motivation	0.848
Science self-efficacy	0.906

Item-to-scale correlation was calculated to estimate the scale validity (Table 2). The average item-to-scale correlation coefficients for teacher strictness and teacher feedback were 0.743 and 0.886, respectively. The average item-to-scale correlation coefficients for motivational beliefs were 0.914, 0.914, 0.789, and 0.774, respectively, for intrinsic motivation, instrumental motivation, achievement motivation, and self-efficacy. A score above.30 indicates internal consistency (Gable, 1986), thus, the scales were all valid.

**TABLE 2:** Item-to-scale correlations of the six constructs.

Teacher strictness		Teacher feedback		Intrinsic motivation		Instrumental motivation		Achievement motivation		Self-efficacy	
Item	Corr.	Item	Corr.	Item	Corr.	Item	Corr.	Item	Corr.	Item	Corr.
TST1	0.691	TFB1	0.841	INT1	0.899	INS1	0.907	ACH1	0.802	SEF1	0.761
TST2	0.769	TFB2	0.888	INT2	0.897	INS2	0.928	ACH2	0.752	SEF2	0.739
TST3	0.760	TFB3	0.912	INT3	0.921	INS3	0.925	ACH3	0.827	SEF3	0.785
TST4	0.753	TFB4	0.906	INT4	0.924	INS4	0.897	ACH4	0.736	SEF4	0.789
		TFB5	0.884	INT5	0.927			ACH5	0.830	SEF5	0.799
										SEF6	0.776
										SEF7	0.776
										SEF8	0.766
Mean	0.743	Mean	0.886	Mean	0.914	Mean	0.914	Mean	0.789	Mean	0.774

Next, the Pearson correlation coefficients of all constructs were estimated to check if they were significantly correlated before performing a structural equation modeling (SEM) analysis. The relationships among teacher strictness, teacher feedback, and students’ motivation beliefs were then further explored using SEM to have an estimation of whether these relationships varied across Western and East Asian learners. Finally, a multi-group analysis was conducted to determine whether such variations in their relationships were statistically significant.

## Results

### Correlation Analyses

To explore the relationship among teacher feedback, teacher strictness, and students’ motivational beliefs, Pearson correlations were performed. As shown in Table 3, the correlation between teacher strictness and teacher feedback was negative and significant for students from Western cultures (*r* = −0.051, *p* < 0.01). The correlations between teacher strictness and motivational beliefs were significantly negative. Teacher strictness was most strongly correlated with intrinsic motivation (*r* = −0.154, *p* < 0.01), followed by instrumental motivation (*r* = −0.094, *p* < 0.01), self-efficacy (*r* = −0.074, *p* < 0.01), and achievement motivation (*r* = −0.026, *p* < 0.01). Teacher feedback was most strongly correlated with intrinsic motivation (*r* = 0.235, *p* < 0.01), followed by instrumental motivation (*r* = 0.167, *p* < 0.01), self-efficacy (*r* = 0.154, *p* < 0.01), and achievement motivation (*r* = 0.149, *p* < 0.01). Using Bonferroni adjusted significance level of.003, the motivational beliefs constructs were significantly and positively correlated with one another (*r* ranged from 0.211 to 0.437).

**TABLE 3:** Correlations among the 6 constructs for Western learners.

	1	2	3	4	5	6
1. Teacher strictness	1					
2. Teacher feedback	−0.051*	1				
3. Intrinsic motivation	−0.154*	0.235*	1			
4. Instrumental motivation	−0.094*	0.167*	0.430*	1		
5. Achievement motivation	−0.026*	0.149*	0.231*	0.211*	1	
6. Self-efficacy	−0.074*	0.154*	0.437*	0.324*	0.216*	1

*Correlation is significant at the adjusted 0.03 level.

As shown in Table 4, for East Asian learners the correlation between teacher strictness and teacher feedback was significantly positive (*r* = 0.069, *p* < 0.01). There were significantly positive correlations between teacher strictness and motivational beliefs. Specifically, achievement motivation (*r* = 0.058, *p* < 0.01) had the strongest correlation with perceived teacher strictness, followed by intrinsic motivation (*r* = 0.046, *p* < 0.01), instrumental motivation (*r* = 0.021, *p* < 0.01), and self-efficacy (*r* = 0.010, *p* = 0.031). The strongest correlation between teacher feedback and students’ motivational beliefs was for intrinsic motivation (*r* = 0.265, *p* < 0.01), followed by instrumental motivation (*r* = 0.247, *p* < 0.01), self-efficacy (*r* = 0.175, *p* < 0.01), and achievement motivation (*r* = 0.136, *p* < 0.01). In addition, the motivational beliefs were significantly and positively correlated with one another at the Bonferroni adjusted 0.003 significance level (*r* ranged from 0.220 to 0.460), except for teacher strictness and self-efficacy.

**TABLE 4:** Correlations among the 6 constructs for East Asian learners.

	1	2	3	4	5	6
1. Teacher strictness	1					
2. Teacher feedback	0.069*	1				
3. Intrinsic motivation	0.046*	0.265*	1			
4. Instrumental motivation	0.021*	0.247*	0.460*	1		
5. Achievement motivation	0.058*	0.136*	0.250*	0.220*	1	
6. Self-efficacy	0.010	0.175*	0.386*	0.289*	0.233*	1

*Correlation is significant at the adjusted 0.03 level.

### SEM Analyses

SEM analyses were performed to further explore the relationships among the variables. We separated Western and East Asian students and used the same model to illustrate the relationships between perceived teacher strictness, teacher feedback, and motivational beliefs.

Multiple fit indices are used for SEM, one of the most common is the ratio of chi-square (χ^2^) statistic (Lehman et al., 2013). However, the value of chi-square statistic is sensitive to sample size (Anderson and Gerbing, 1988) as a large sample size may generate a significant chi-square result with minor discrepancies. Given a rather large sample size of the current study (*n* = 89,869), it will be more robust to adopt multiple goodness-of-fit indices, including CFI, PNFI, and RMSEA.

**FIGURE 1:**
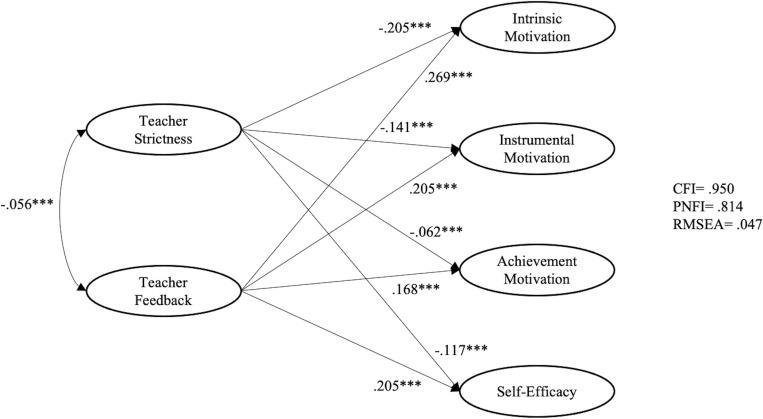
Relationships among teacher feedback, teacher strictness, and motivational beliefs for Western students.

As shown in Figure 1, for students from Western cultures, teacher strictness had negative and significant effects on all constructs of motivational beliefs, including intrinsic motivation (β = −0.205, *p* < 0.01), instrumental motivation (β = −0.141, *p* < 0.01), achievement motivation (β = −0.062, *p* < 0.01), and self-efficacy (β = −0.117, *p* < 0.01). Teacher feedback had positive and significant effects on intrinsic motivation (β = 0.269, *p* < 0.01), instrumental motivation (β = 0.205, *p* < 0.01), achievement motivation (β = 0.168, *p* < 0.01), and self-efficacy (β = 0.205, *p* < 0.01). As suggested by Hu and Bentler (1999), the indices of this model (CFI = 0.950; PNFI = 0.814; RMSEA = 0.047) indicate an excellent fit to the data.

**FIGURE 2:**
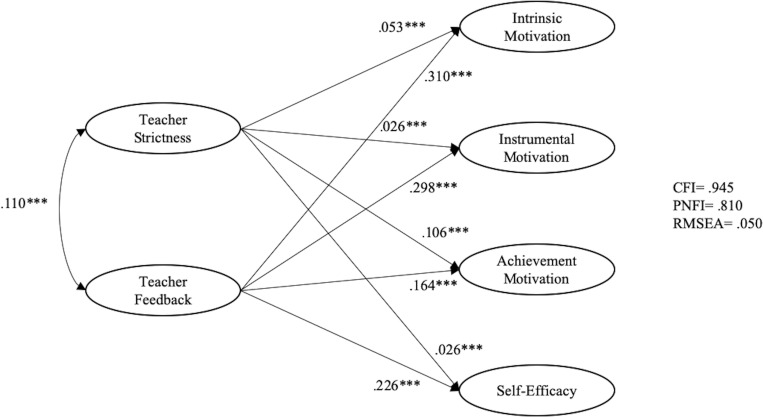
Relationships among teacher feedback, teacher strictness, and motivational beliefs for East Asian students.

Figure 2 shows the effects of teacher strictness and teacher feedback on East Asian students’ motivational beliefs. Teacher strictness had a weak but positive and significant effect on intrinsic motivation (β = 0.053, *p* < 0.01), instrumental motivation (β = 0.026, *p* < 0.01), achievement motivation (β = 0.106, *p* < 0.01), and self-efficacy (β = 0.026, *p* < 0.01). Teacher feedback also had a positive and significant effect on intrinsic motivation (β = 0.310, *p* < 0.01), instrumental motivation (β = 0.298, *p* < 0.01), achievement motivation (β = 0.164, *p* < 0.01), and self-efficacy (β = 0.226, *p* < 0.01). The model fit indices (CFI = 0.945; PNFI = 0.810; RMSEA = 0.050) indicate an excellent fit to the data.

Since considerable differences were found between Figures 1, 2 in the relationships between (i) teacher strictness and motivational beliefs and (ii) teacher feedback and strictness, multi-group analysis was performed to examine if such differences were statistically significant across Western and East Asian learners. Four models were imposed, with good model fits (Table 5). First, no equality constraints were imposed in the baseline model (M1). Following M1, M2 was imposed by forcing equal constraints on measurement weights. The change in CFI between the models was.002, which is below the.01 threshold for invariance as suggested by Cheung and Rensvold (2002). Equality constraints were further imposed on measurement weights and structural weights (M3). The change in CFI was 0.015, which is above the 0.01 threshold. For the fourth model, equality constraints were imposed on the measurement weights, structural weights, and structural covariance. The change in CFI was 0.001. All these findings indicated that there exists significant difference in the structural weights between Western and East Asian learners.

**TABLE 5:** Summary of goodness-fit statistics for tests of multigroup invariance across the Western and East Asian students.

	RMSEA	PNFI	CFI	Change in CFI
M1 Baseline model (no constraints imposed)	0.034	0.812	0.948	–
M2 Invariant measurement weights	0.034	0.834	0.946	0.002
M3 Invariant measurement weights and structural weights	0.038	0.857	0.931	0.015
M4 Invariant measurement weights, structural weights, and structural covariances	0.038	0.860	0.930	0.001

## Discussion

### Teacher Feedback, Teacher Strictness, and Students’ Motivational Beliefs

As indicated in Figures 1, 2, teacher feedback had significant positive association with both Western and East Asian students’ motivational beliefs. For Western students, teacher feedback had the greatest association with intrinsic motivation, followed by self-efficacy, instrumental motivation, and achievement motivation. For East Asian students, teacher feedback had the greatest association with intrinsic motivation, followed by instrumental motivation, self-efficacy, and achievement motivation. This finding is in line with previous findings that teacher feedback is positively related to students’ motivational beliefs (e.g., Hamidun et al., 2012; Pat-El et al., 2012).

It is interesting to note that while teacher strictness is negatively related to Western students’ motivational beliefs, its relations were significantly positive for Eastern students. This finding echoes the results of Maulana et al. (2011) study in Indonesia that the more teachers exhibit dominance and cooperation, the more students are motivated to engage in learning. However, these results are opposite to findings from the West (e.g., Brekelmans et al., 1993; Wubbels and Brekelmans, 2005).

The disparities in the associations between teacher strictness and students’ motivations to learn might be caused by different social and cultural factors in interpreting the roles and expectations of teachers and students. Eastern Asia is characterized by Confucian heritage cultures, such as China, Korea, and Japan (e.g., Kim, 2009; Min, 2016; Xiao and Hu, 2019), which often practice a large power distance (i.e., high acceptance of an unequal power distribution) (Hofstede et al., 2010). Under the influence of Confucianism, Eastern societies often emphasize obedience to authority figures and compliance with group interests (Chang et al., 2011), whereas in Western cultures, the power distance between social members is relatively small (i.e., superiority over others is often considered unacceptable) (Hofstede et al., 2010). Hence, in Western societies, individual thinking and interest are valued, and individual differences are appreciated (Hofstede et al., 2010).

Cultural differences also exist in the expectations and roles of teachers and students. In the Confucian context, teachers are expected to become the models to help students to realize their good natures, or to introduce models for students to emulate (Shim, 2008). This suggests teachers have significant influences and controls in students’ learning as means to cultivate their excellence. Most classrooms in contemporary East Asia are featured as teacher-centered practices. A good teacher is considered as being able to strictly control classroom processes (Zhu et al., 2010; Sun et al., 2018), while a good student is someone who respects and is obedient to their teachers (Zhou et al., 2012). Hence, East Asian students tend to have high expectations and acceptance of teachers’ strict or dominant behaviors in class (Hofstede et al., 2010; Wei et al., 2015). In contrast, some of the philosophical underpinnings in Western cultures (such as the philosophy of Socrates) emphasize that teachers should not simply pass on knowledge but also investigate and explore with students together, and that students should be able to think and express their own views, and teachers could correct their views through conversations with them (Shim, 2008). With such philosophical roots, classrooms in Western cultures are often featured as student-centered processes. Teachers are valued for supporting students’ autonomy, freedom, and choices, treating each student as a unique individual, as well as maintaining companionate communications with students, while students are valued for demonstrating autonomy and independence in their learning (Hofstede et al., 2010; Chang et al., 2011).

Given the differences in social norms and cultural roots as discussed above, teacher strictness may be more acceptable to East Asian students because it meets their expectations and aligns with the cultural and social values of Eastern societies, which in turn has no negative connections with these students’ motivational beliefs. In contrast, Western students may consider teacher strictness to interfere with their freedom, independence, and autonomy in learning (Chan and Rao, 2010). Therefore, when they feel that their teachers are stricter, their learning motivations and efficacy will be reduced.

The positive influence of teacher strictness on East Asian students’ motivation can be further explained by the deeply rooted beliefs that teacher strictness is an indication of high expectations from East Asian cultures (Watkins and Biggs, 2001). There is an old Chinese saying, “a strict teacher produces outstanding students” (
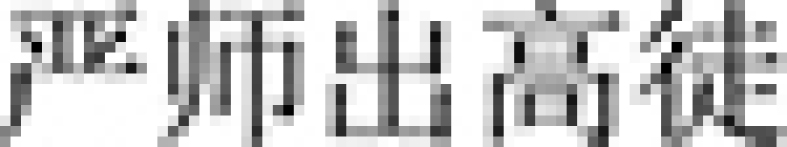
). This implies that if a teacher is stricter with a student, he or she has high expectations of that student. Therefore, when a Chinese student perceives teacher strictness, the student may consider it to be recognition of the importance of his or her learning, and so be more confident and motivated to learn. This inference seems to be supported by the positive relationship between teacher strictness and teacher feedback in the East Asian culture revealed in this study. When an East Asian teacher is stricter with some students, more feedback will be provided to these students, which may be commonly regarded by teachers and students as a sign of high expectations. In contrast, but not surprisingly, the relationship between teacher strictness and teacher feedback in the Western culture was found to be weak and negative.

### Teacher Strictness and Teacher Feedback

In classroom learning environment, close correlations between teacher interpersonal and teaching behaviors were reported in two previous studies (Cheng and Wan, 2017; Wan and Cheng, 2019). In these two studies, teaching behaviors included assigning challenging tasks to students, stimulating multiple perspectives and encouraging students to communicate with each other, while interpersonal behaviors included sharing control with students and allowing skeptical voice. However, in this study, although the significant correlation was found between teacher strictness and teacher feedback in the East Asian context, their correlation coefficient was rather weak in the Western context. Compared with the two previous studies of classroom learning environment, the correlation coefficients generated in this study were rather small, which indicates the strengths of the correlations between different teacher interpersonal and teaching behaviors may be various.

This study further revealed that with the significant correlation between teacher interpersonal and teaching behaviors (i.e., teacher strictness and teacher feedback), there might still exist cultural differences. The positive significant correlation between teacher strictness and teacher feedback in the East Asian context may be interpreted by the examination-oriented culture. It is well-known that examination culture is prevailing in China and other East Asian regions (Cheng, 2004; Zhan and Wan, 2010). Within such culture, if a teacher is stricter with their students, it implies that he/she has a higher expectation for their students’ examination performance, which may cause them to give more feedback to their students so as to enhance their performance. Therefore, it is logical to have a significant and positive correlation between East Asian teacher strictness and feedback. In contrast, in the regions where the examination-oriented culture is not dominant, such a correlation between teacher strictness and teacher feedback may not exist because the connection cannot be established between teacher strictness and a high expectation for students’ examination performance.

### Implications, Limitations, and Future Studies

Echoing the argument made by Sun et al. (2019) that findings from Western countries may not be directly generalizable to Eastern countries given the multi-layered differences that exist in Western and Eastern cultures, the current study has the following implications. First, although both Western and East Asian teachers should consider giving more constructive feedback to their students, Western teachers should be careful when doing some behaviors that may be perceived as strict by Western students. At the same time, East Asian teachers can be a bit strict to their students, but they should pay attention to the reaction of their students (especially low performing students) since over-strictness may harm their learning motivations and efficacy. Second, there has been a growing trend of pedagogical reform in East Asian countries in recent years, and the reforms usually include adopting sophisticated research findings from mostly Western countries into teaching practice. However, teachers should be cautious and selective when adopting teaching strategies in accordance with local cultural environments. Third, taking a culturally responsive classroom management perspective (Weinstein et al., 2004), it is important for teachers, especially those from Western countries who work in schools with populations of students from multicultural backgrounds, to be conscious of the potential for different interpretations of interpersonal behaviors in different cultures.

A number of limitations should be acknowledged in the current study. The first limitation lays on insufficient information that could be provided by pre-collected PISA data. The current study revealed a significant difference in the effects of teacher behaviors on students’ motivational beliefs between East Asian and Western learners. However, given the pre-collected nature of PISA data, there lacks data to further reveal why such difference exists. Second, teachers’ behaviors and students’ motivational beliefs were retrieved from students’ self-reported perceptions, and the results might be limited by self-reported data.

Stemming from current findings, further research should be conducted to explore the complex connections between teacher strictness and teacher feedback utilizing multiple methods. In addition, the current research is a brief report that only make a concise investigation of the issues by comparing the differences between East Asian and Western students. Further investigations may examine not only cross-cultural differences but also intracultural differences using comparative data, such as TIMSS and PIRLS. The comparisons in terms of gender or students’ achievement are interesting directions for future research as well.

## Data Availability Statement

Publicly available datasets were analyzed in this study. This data can be found here: http://www.oecd.org/pisa/data/.

## Author Contributions

YJ: analysis and interpretation of data for the work and drafting of the work. C-KL: design of the work and revising the draft. ZW: analysis and interpretation of data for the work and revising the draft. JC: analysis and interpretation of data for the work. All authors contributed to the article and approved the submitted version.

## Conflict of Interest

The authors declare that the research was conducted in the absence of any commercial or financial relationships that could be construed as a potential conflict of interest. The reviewer MN declared a shared affiliation with the authors, to the handling editor at the time of review.

## Footnotes

1 http://www.oecd.org/pisa/data/

2 http://www.oecd.org/pisa/data/2015database/

## Appendix

**TABLE A1:** Items of measurements.

Construct	Name	Content	Scaling
Teacher strictness	TST1	Teachers called on me less often than they called on other students.	1 = never or almost never; 2 = a few times a year; 3 = a few times a month; 4 = once a week or more
	TST2	Teachers graded me harder than they graded other students.	
	TST3	Teachers disciplined me more harshly than other students.	
	TST4	Teachers gave me the impression that they think I am less smart than I really am.	
Teacher feedback	TFB1	The teacher tells me how I am performing in this course.	1 = never or almost never; 2 = some lessons; 3 = many lessons; 4 = every lesson or almost every lesson.
	TFB2	The teacher gives me feedback on my strengths in the science subject.	
	TFB3	The teacher tells me in which areas I can still improve.	
	TFB4	The teacher tells me how I can improve my performance.	
	TFB5	The teacher advises me on how to reach my learning goals.	
Intrinsic motivation	INT1	I generally have fun when I am learning science topics.	1 = strongly disagree; 2 = disagree; 3 = agree; 4 = strongly agree
	INT2	I like reading about science.	
	INT3	I am happy working on science topics.	
	INT4	I enjoy acquiring new knowledge in science.	
	INT5	I am interested in learning about science.	
Instrumental motivation	INS1	Making an effort in my science subject(s) is worth it because this will help me in the work I want to do later on.	
	INS2	What I learn in my science subject(s) is important for me because I need this for what I want to do later on.	
	INS3	Studying my science subject(s) is worthwhile for me because what I learn will improve my career prospects.	
	INS4	Many things I learn in my science subject(s) will help me to get a job.	
Achievement motivation	ACH1	I want top grades in most or all of my courses.	
	ACH2	I want to be able to select from among the best opportunities available when I graduate.	
	ACH3	I want to be the best, whatever I do.	
	ACH4	I see myself as an ambitious person.	
	ACH5	I want to be one of the best students in my class.	
Self-efficacy	SEF1	Recognize the science question that underlies a newspaper report on a health issue.	1 = I couldn’t so this; 2 = I could struggle to do this on my own; 3 = I could do this with a bit of effort; 4 = I could do this easily.
	SEF2	Explain why earthquakes occur more frequently in some areas than in others.	
	SEF3	Describe the role of antibiotics in the treatment of disease.	
	SEF4	Identify the science question associated with the disposal of garbage.	
	SEF5	Predict how changes to an environment will affect the survival of certain species.	
	SEF6	Interpret the scientific information provided on the labeling of food items.	
	SEF7	Discuss how new evidence can lead you to change your understanding about the possibility of life on Mars	
	SEF8	Identify the better of two explanations for the formation of acid rain.	
